# sCD4-17b bifunctional protein: Extremely broad and potent neutralization of HIV-1 Env pseudotyped viruses from genetically diverse primary isolates

**DOI:** 10.1186/1742-4690-7-11

**Published:** 2010-02-16

**Authors:** Laurel A Lagenaur, Vadim A Villarroel, Virgilio Bundoc, Barna Dey, Edward A Berger

**Affiliations:** 1 Laboratory of Viral Diseases, National Institute of Allergy and Infectious Diseases, National Institutes of Health, Bethesda, MD 20892, USA

## Abstract

**Background:**

We previously described a potent recombinant HIV-1 neutralizing protein, sCD4-17b, composed of soluble CD4 attached via a flexible polypeptide linker to an SCFv of the 17b human monoclonal antibody directed against the highly conserved CD4-induced bridging sheet of gp120 involved in coreceptor binding. The sCD4 moiety of the bifunctional protein binds to gp120 on free virions, thereby enabling the 17b SCFv moiety to bind and block the gp120/coreceptor interaction required for entry. The previous studies using the MAGI-CCR5 assay system indicated that sCD4-17b (in concentrated cell culture medium, or partially purified) potently neutralized several genetically diverse HIIV-1 primary isolates; however, at the concentrations tested it was ineffective against several other strains despite the conservation of binding sites for both CD4 and 17b. To address this puzzle, we designed variants of sCD4-17b with different linker lengths, and tested the neutralizing activities of the immunoaffinity purified proteins over a broader concentration range against a large number of genetically diverse HIV-1 primary isolates, using the TZM-bl Env pseudotype assay system. We also examined the sCD4-17b sensitivities of isogenic viruses generated from different producer cell types.

**Results:**

We observed that immunoaffinity purified sCD4-17b effectively neutralized HIV-1 pseudotypes, including those from HIV-1 isolates previously found to be relatively insensitive in the MAGI-CCR5 assay. The potencies were equivalent for the original construct and a variant with a longer linker, as observed with both pseudotype particles and infectious virions; by contrast, a construct with a linker too short to enable simultaneous binding of the sCD4 and 17b SCFv moieties was much less effective. sCD4-17b displayed potent neutralizing activity against 100% of nearly 4 dozen HIV-1 primary isolates from diverse genetic subtypes (clades A, B, C, D, F, and circulating recombinant forms AE and AG). The neutralization breadth and potency were superior to what have been reported for the broadly neutralizing monoclonal antibodies IgG b12, 2G12, 2F5, and 4E10. The activity of sCD4-17b was found to be similar against isogenic virus particles from infectious molecular clones derived either directly from the transfected producer cell line or after a single passage through PBMCs; this contrasted with the monoclonal antibodies, which were less potent against the PMBC-passaged viruses.

**Conclusions:**

The results highlight the extremely potent and broad neutralizing activity of sCD4-17b against genetically diverse HIV-1 primary isolates. The bifunctional protein has potential applications for antiviral approaches to combat HIV infection.

## Background

The human immunodeficiency virus (HIV) envelope glycoprotein (Env) mediates virion entry into target cells by orchestrating sequential binding of the gp120 subunit to receptors on the target cell surface, first to CD4, then to the coreceptor (chemokine receptor CCR5 or CXCR4); receptor binding then activates the Env gp41 subunit to promote direct fusion between the virion and plasma membranes [1-3]. The binding sites for both CD4 and coreceptor contain determinants that are highly conserved, not only within the quasispecies present in the infected individual, but also across the wide genetic diversity of HIV-1 variants found globally. Env has evolved a multilayered structural strategy to protect these critical conserved elements, thereby allowing chronic replication to continue in the face of a humoral antibody response that might otherwise be neutralizing [4-8]. Particular attention has been given to a "conformational masking" mechanism [9] whereby the highly conserved "bridging sheet" of gp120 [10,11], a critical component of the coreceptor binding site [12,13], is hidden or unformed on free virions, and becomes exposed/formed/stabilized only after gp120 undergoes major conformation changes induced by CD4 binding [9,14,15].

These structural complexities have profound implications for HIV neutralization by antibody. The immune system is capable of eliciting high titer antibody responses against the conserved CD4-induced bridging sheet, both during natural infection [16] and in response to immunization, particularly with appropriately engineered gp120 derivatives [17-19]. Several human monoclonal antibodies (MAbs) directed against the bridging sheet have been derived from B cells of infected individuals [20-24]. These MAbs, of which 17b is an extensively studied prototype, are broadly cross-reactive with gp120 molecules from widely diverse HIV-1 primary isolates. Indeed, the first X-ray crystallographic structures of gp120 were solved for a trimolecular complex containing a gp120 "core" bound to a soluble CD4 (sCD4) construct containing the first 2 extracellular domains and the 17b Fab [10,11]. While antibodies against the bridging sheet bind avidly to gp120-CD4 complexes and block their interaction with coreceptor [22,23,25,26], they are weakly neutralizing for HIV-1 primary isolates because the epitopes are poorly exposed or unformed/unstable on the virion prior to its engagement with CD4 [22,27]. An additional layer of Env protection is afforded by the steric hindrance when the virion is bound to CD4 on the target cell surface; the narrow space between the virion and cell membranes impairs access of an intact IgG molecule to the CD4-induced bridging sheet [28]. Thus a particularly tempting but vexing challenge arises, namely how to design a strategy whereby an anti-bridging sheet antibody can access its highly conserved epitope on the free virion prior to its engagement with CD4 on the target cell, thus neutralizing infectivity for genetically diverse HIV-1 variants.

We previously reported the design of a bifunctional HIV-1 neutralizing protein that exploits the two-step receptor interaction mechanism to circumvent the conformational masking and steric hindrance mechanisms that impede antibody access to the conserved bridging sheet on gp120 [29]. sCD4-17b is a recombinant single chain protein consisting of the first 2 domains of human CD4 attached by a flexible polypeptide linker to a single chain variable region construct (SCFv) of the 17b MAb. The sCD4 moiety binds to gp120 on free virions and induces the 17b epitope; binding of the 17b SCFv moiety then blocks coreceptor interaction, thereby neutralizing infectivity. We reported that sCD4-17b potently neutralized several HIV-1 primary isolates of approximately a dozen tested; however, nearly half were resistant, despite the highly conserved nature of both the CD4 and 17b binding sites. We speculated on plausible reasons for the disappointingly limited neutralization breadth, and proposed several experimental approaches to test these explanations and possibly resolve the problem.

In the present report, we expressed and purified variant forms of sCD4-17b and employed a widely used high throughput assay to measure neutralization of lentiviral particles pseudotyped with Envs from a large number of genetically diverse HIV-1 primary isolates. Our results are highly favorable, with potent neutralization of virtually 100% of the nearly 4 dozen pseudotypes tested. The neutralization breadth was considerably greater than that reported for the well-characterized broadly neutralizing MAbs IgG b12, 2G12, 2F5 and 4E10. Moreover, we found that sensitivity to sCD4-17b was relatively independent of the cellular source from which the virions were produced, unlike the above-mentioned MAbs whose efficacy was significantly influenced, as previously reported by others [30]. These results reinvigorate prospects for practical applications of sCD4-17b in efforts to combat the HIV pandemic.

## Methods

### Design and expression of sCD4-17b variants and related protein constructs

As previously described [29], sCD4-17b contains the first two domains of human CD4 (residues 1-183) attached by a flexible polypeptide linker (designated L1) to an SCFv of the 17b human MAb (V_H _attached to V_L _by the 5 amino acid linker G_4_S, designated L2). In the original construct, the L1 linker contained 35 amino acids (seven repeats of the G_4_S motif). The alternate sCD4-17b variants described in the present study are herein designated according to the number of amino acids in the L1 linker (in each case, composed of the associated number of G_4_S repeats). The constructs described here are sCD4-35-17b (as in [29]), sCD4-40-17b, and sCD4-5-17b. To enhance expression and subsequent purification, all variants contained the N-terminal leader sequence of human Ig kappa light chain, and a 9 amino acid C-terminal epitope tag derived from the intracellular C-terminus of bovine rhodopsin (designated C9, with the following sequence: TETSQVAPA) that is recognized by the rho 1D4 MAb [31] (herein referred to as 1D4). Constructs representing the individual moieties (sCD4 and 17b scFV) were also prepared, each containing the same N-terminal leader sequence and C-terminal C9 epitope tag.

The DNA constructs were cloned first by PCR using a Topo TA vector (Invitrogen), then digested with SalI and NotI and ligated into SalI and NotI sites in the plasmid vector VRC8400 pCMV/R (a generous donation of G. Nabel, NIH Vaccine Research Center) containing the enhanced human cytomegalovirus promoter CMV/R [32]. Plasmids were transformed into *E. coli *One Shot Top10 cells (Invitrogen) and grown under kanamycin selection. DNA was prepared using a Plasmid Maxi Kit (Qiagen). Proteins were expressed by transient transfection of re-adherent FreeSyle 293F cells (Invitrogen) using Fugene (Roche) according to manufacturer's instructions. Briefly, each 162 cm^2 ^flask was seeded with ~4 × 10^6 ^cells in DMEM containing 10% FCS, 24 hr prior to transfection. The morning of transfection DMEM was removed and replaced with serum-free FreeStyle Medium (Invitrogen). Transfection mixtures were prepared containing 10 μg of plasmid DNA, 100 μl Fugene in 800 μl FreeStyle Medium and incubated 30 min at room temperature. Cells were then transfected using polypropylene tips to deliver the DNA to the monolayer and incubated for 5 days at 37°C. Culture supernatants were harvested and centrifuged at 3500 RPM for 10 minutes to remove cell debris. Supernatants were concentrated 10× with filters (Millipore, 30 kDa cutoff for sCD4-17b and 10 kDa cutoff for the sCD4 and 17b SCFv individual proteins), dialyzed against PBS pH 7.4, and either used immediately for purification or frozen and stored at -80°C until further use.

### Protein purification and analysis

The various sCD4-17b variants (and the proteins representing the individual sCD4 and 17b SCFv moieties) were purified from the 10× concentrated supernatants using a single step immuno-affinity procedure based on binding of the C9 epitope tag to the 1D4 MAb [33]. Briefly, CNBr-activated Sepharose 4B (GE Healthcare) was prepared according to the manufacturer's instructions and washed in 1 mM HCl. 1D4 murine MAb (anti-C9, purchased from Flintbox, University of British Columbia) was coupled in batch to the activated Sepharose 4B in 0.1 M NaHCO_3 _containing 0.5 M NaCl at a concentration of 5-10 mg protein/ml medium. The mixture was rotated end-over-end overnight at 4°C. Active groups were blocked with 0.1 M Tris-HCl buffer, pH 8.0 for 2 hours, and the beads were washed in three cycles of alternating pH, 0.1 M acetic acid/sodium acetate, pH 4.0 containing 0.5 M NaCl and 0.1 M Tris-HCl, pH 8.0 containing 0.5 M NaCl. Concentrated media supernatants containing sCD4-17b proteins (50-100 μg/ml media) were diluted in Immunoaffinity Buffer [100 mM (NH_4_)_2_SO_4_, 20 mM Tris pH 8.0, 2% glycerol] and then bound in batch to the Sepharose-1D4 overnight at 4°C. Approximately 5 ml of Sepharose-1D4 mixture were then loaded onto single use columns (BioRAD) and washed four times with 5 ml of Immunoaffinity Buffer, followed by a fifth wash with 5 ml Immunoaffinity Buffer supplemented with 500 mM MgCl_2_. Bound protein was competitively eluted using five elutions with 5 ml Immunoaffinity Buffer-500 mM MgCL_2 _containing 250-500 μM C9 peptide (95-98% purity, American Peptide Company). Alternatively in some cases, C9 peptide elution was performed in conjunction with low pH using two elutions with 250 μM C9 peptide in 100 mM glycine HCL, pH 2.7; fractions were collected into tubes containing equal volumes of Tris HCl pH 9.0 to neutralize the eluates. The final material was concentrated in BSA passivated filters (Millipore, as described above) and dialyzed against PBS, pH 7.4. Protein concentrations were determined by quantitative immunoblot analysis of serial dilutions of protein samples using the Odyssey Imager (Li-Cor Biosciences) compared to a 2-domain sCD4 protein standard of known concentration (provided by S Leow, Upjohn). Preparations of purified sCD4-17b ranging from ~10-25 mg/ml (corresponding to ~200-500 μM) were stored at 4°C.

Proteins were analyzed by reducing SDS-PAGE combined with Coomassie Blue staining or Western blot analysis. For Western blots, proteins were resolved on 4-12% Bis-Tris gels (Invitrogen), then transferred to PDVF membranes using the IBlot Gel Transfer System (Invitrogen). Primary antibody [sheep polyclonal anti-CD4, NIAID AIDS Research and Reference Reagent Program (ARRRP)], 1:5000; or 1D4 murine anti-C9 MAb, 1:1000) was diluted in Odyssey Blocking Buffer (Li-Cor Biosciences) and incubated on the blots for 1 hr at room temperature with gentle shaking. After three vigorous washes (PBS, pH 7.4 with 0.2% Tween 20, Sigma), the blots were incubated with secondary antibody (anti-sheep or anti-mouse immunoglobulin, IRDyes, Li-Cor Biosciences) diluted 1:1000 in Odyssey Blocking Buffer and incubated in a light resistant container for 45-60 min at room temperature. The blots were given four vigorous washes in wash buffer and a final wash in PBS. Proteins were visualized using the Odyssey Imager (Li-Cor Biosciences).

### HIV-1 particle preparation

Lentivirus particles pseudotyped with the indicated HIV-1 Env were prepared as described [34]. Briefly, 293T cells (human kidney fibroblast cell line) were cultured in DMEM with 10% FCS and 0.0002% plasmocin. T225 flasks were seeded with 6 × 10^6 ^cells. The following day, flasks were transfected with 30 μg backbone plasmid DNA and 10 μg Env plasmid DNA, and 120 μl Fugene reagent in 1.2 ml FreeStyle medium. The mixture was incubated at room temperature for 30 min, then applied to the cell monolayer with a polypropylene pipette tip. The cells were incubated overnight at 37°C in 5% CO2, after which the medium was removed and 35 ml of fresh DMEM-10% was added. At 48 hrs post-transfection, the supernatant was removed and filtered through a 0.45 μM filter (Millipore). The supernatant was then divided into 1 ml aliquots and stored frozen at -80°C. Assays were performed with samples that had been frozen/thawed only a single time.

Expression plasmids encoding most of the Envs from clades A, B, and C were obtained from ARRRP; vectors are indicated in the corresponding data sheets. Expression plasmids for Envs from 92RW020 and DJ263.8 (both clade A) as well as YU2 and Ba-L (both clade B) were kindly provided by John Mascola (Vaccine Research Center, NIH). Functional pseudotype particles were generously donated by Vicky Polonis (Walter Reed Army Institute of Research) and Sodsai Tovanabutra (Henry M. Jackson Foundation) for some clade A Envs as well as for Envs from clade D and the circulating recombinant forms AE and AG (see Figure Legends). For isolates 91US054 (clade B), 93IN905 (clade C), and 93BR029 (clade F), the Env genes were amplified by PCR from PBMC cultures infected with the corresponding primary isolates (obtained from ARRRP) using conserved primers; the products were cloned first into a TOPO TA vector (Invitrogen), then into VRC8400 pCMV/R (Not1 and Sal1 sites) to generate pseudotypes as described above.

A series of experiments was performed with virus particles derived from infectious molecular clones (IMCs) from the BL01 and 89.6 isolates (both clade B). For each, two types of particles (generously donated by John Mascola and Mark Louder, Vaccine Research Center, NIH) were employed: particles derived directly from 293T cells transfected with the corresponding plasmids, and particles derived by single passage of the 293T-derived viruses through mitogen-activated PBMCs [30].

### HIV-1 neutralization assays

The major neutralization assay employed herein was analysis of HIV-1 Env pseudotype infection of TZM-bl (JC53bl-13) cells [34]. This single cycle assay involves measurement of luciferase activity in lysates of cells containing the firefly luciferase gene linked to the HIV-1 LTR, dependent on entry of the pseudovirus particle. Briefly, serial dilutions of the indicated agents were made in PBS pH 7.4 in a 96 well plate, pseudovirus particles were then added to the agents and incubated for 30 min. TZM-bl cells were trypsinized and added to each well, and the plates were incubated for 48 hrs at 37°C in 5% CO2. The cells were then lysed with Bright-Glo Luciferase reagent (Promega) and luciferase activity was measured using the Clarity luminometer (Biotek). In the case of "live virus", assays were performed in the same manner except that the cells were lysed with cell culture lysis buffer (Promega), prior to addition of Bright-Glo reagent. All pseudotype preparations were titered by measuring luciferase activity obtained with serial dilutions of the stock preparation. Neutralization experiments were performed with viral inputs of 50-200 TCID_50 _based on the cytopathic effects of particular pseudotypes. IC_50 _values were determined with Prism 5 (GraphPad Software): nonlinear regression (curve fit); log(inhibitor) versus response - variable slope (four parameters); least squares (ordinary fit); unknowns interpolated from standard curve (95% confidence interval). Activities are expressed as direct measurement of Relative Luminescence Units (RLU), or in some cases as % of the designated control.

A small number of experiments were performed using the MAGI-CCR5 assay [35] as previously described [29]. Briefly, 20 μl virus dilutions (expected to generate approximately 200 blue cells per 10^4 ^MAGI-CCR5 cells) were preincubated with 30 μl serial dilutions (in PBS, pH 7.4) of sCD4-17b protein for 30 min at 37°C; 20 μg/ml final concentration of DEAE-dextran was then added to this mix and the contents were transferred to individual wells of a 96-well plate containing the MAGI-CCR5 cells. After 2 hours, 150 μl of DMEM-10% FCS was added to each well and the plate was incubated for 48 hours before fixing and staining the cells for microscopic counting of blue nuclei.

## Results

Our previous studies [29] analyzed a single sCD4-17b construct produced in modest quantities and assayed mainly in the context of concentrated conditioned medium containing the secreted protein. The neutralization assay employed infectious HIV-1 virions from several primary isolates, using the MAGI-CCR5 system based on microscopic visualization and counting of infected cells after *in situ *staining for β-galactosidase-positive nuclei [35,36]. To expand upon these initial studies, in the present report, we employed an efficient mammalian transient transfection system to produce mg quantities of several sCD4-17b variant constructs and related proteins, coupled with single-step immunoaffinity purification. For HIV-1 neutralization, we used the single round TZM-bl/Env pseudotype assay method, in which the firefly luciferase gene linked to the HIV-1 LTR is activated upon virion entry [34]. This high throughput system has many desirable features for neutralization assays, and has been adopted as a major component for evaluating plasma antibodies generated during natural infection and vaccine trials [37,38], as well for characterizing the breadth of neutralization by various MAbs [39-41].

### Expression and purification of secreted sCD4-17b variants and related proteins

We previously speculated that a possible explanation for the observed limited breadth of the original sCD4-17b construct was that for some Envs, the flexible L1 linker connecting the sCD4 and 17b SCFv moieties might have been insufficiently long to enable simultaneous binding of both components to the same gp120 subunit; differences in the size and conformation of variable loops (which were not present in the gp120 core used for X-ray crystallographic structure determinations) as well as possible differences in orientation of the binding sites for CD4 and 17b were offered as possible contributing factors [29]. To extend the earlier studies, we designed sCD4-17b variants with different L1 linker lengths. The proteins are designated herein with a number representing the total number of amino acid residues in the L1 linker (composed of repeats of the G_4_S motif). Based on the reported X-ray crystallographic analyses of ternary complexes containing gp120 core proteins bound to 2 domain sCD4 and the 17b Fab [10,11], the flexible L1 linker connecting the sCD4 and 17b SCFv moieties must span an atomic distance of 60 Å. Our previous studies [29] were performed with a construct with an L1 linker consisting of 7 G_4_S repeats (herein designated sCD4-*35*-17b); this length was predicted to be sufficiently long to allow simultaneous binding of both moieties to a single gp120 subunit. In the present study, we wished to test whether a construct with a longer linker (sCD4-*40*-17b) could overcome the previously observed limited breadth; as a negative control, we also produced a construct with an L1 linker predicted to be far too short to allow simultaneous binding (sCD4-*5*-17b).

**Figure 1:**
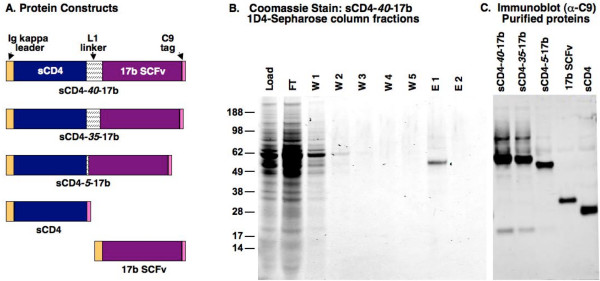
**Design and purification of sCD4-17b constructs and related proteins**. **A) **Schematic representation of three sCD4-17b constructs with different L1 linkers, with the total number of L1 amino acids indicated in the construct name (in each case consisting of the appropriate number of repeats of the G_4_S motif). Also shown are the constructs representing the individual components sCD4 and 17b SCFv. All constructs include the Ig kappa light chain leader sequence at the amino terminus, and the C9 epitope tag at the carboxy terminus. **B) **Immunoaffinity purification of sCD4-*40*-17b, as analyzed by Coomassie Blue staining of reducing SDS-PAGE gels (10 μl per lane for each sample). In this example, C9 peptide elution was performed in conjunction with low pH. The fractions analyzed were the initial concentrated media supernatant (load), flow-through (FT), the 5 wash fractions (W1-W5) and the two elution fractions (E1, E2). Numbers on the left indicate molecular weight markers (kDa). **C) **Western blot analysis of purified preparations of the indicated sCD4-17b proteins as well as the 17b SCFv and sCD4 proteins (10 μl per lane for each sample). The 1D4 MAb directed against the C-terminal tag on each protein was used for detection.

The sCD4-17b variants, as well proteins representing the corresponding individual sCD4 and 17b SCFv moieties, are depicted in Fig. 1A. In all cases the constructs were engineered with an N-terminal Ig kappa secretion leader sequence (in place of the native CD4 leader sequence) as well as a C-terminal C9 epitope tag (in place of the previous 6-his tag) for single-step immunoaffinity purification from concentrated cell culture supernatants using the 1D4 MAb conjugated to Sepharose 4B beads. These two modifications were found to increase by several fold the amounts of the engineered proteins secreted into the medium (data not shown). We employed an efficient mammalian expression system involving transient transfection of 293F cells with plasmids containing an enhanced human cytomegalovirus promoter. The amounts of sCD4-17b secreted into the culture supernatants typically ranged between 5-8 μg/ml.

An example of immunoaffinity purification is shown for the sCD4-*40*-17b protein (Fig. 1B). Coomassie blue staining of reducing SDS-PAGE gels demonstrated that the expressed protein was only a minor component in the initial concentrated supernatant loaded onto the 1D4-Sepharose beads; it was the major single band in the first C9 peptide eluate fraction (E1), with mobility consistent with the expected 51 kDa. Immunoblot analysis (not shown) indicated that only minimal amounts of the protein were detected in the flow through and wash fractions, confirming the efficiency of this single-step immunoaffinity purification system. The other sCD4-17b variants and the corresponding proteins representing the individual moieties were expressed and purified in similar fashion. Immunoblot analysis (Fig. 1C) verified that each purified protein migrated on reducing SDS-PAGE gels at the corresponding expected mobility. We also observed that purified sCD4-17b proteins migrated on non-reducing gels as monomers (~51 kDa, data not shown).

### Effects of the L1 linker length of sCD4-17b and HIV-1 virus particle types on neutralization

**Figure 2:**
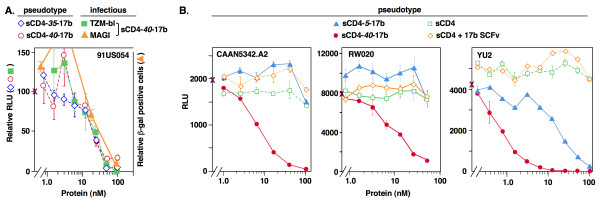
**HIV-1 neutralization of various isolates by different sCD4-17b constructs and related proteins**. Assays were performed using the TZM-bl system or where indicated, with the MAGI-CCR5 system. Dose-response analyses were performed with the indicated proteins and HIV-1 Env pseudotypes or infectious virus, as indicated by the symbols above the graphs and the names within the graphs. Each point represents the mean of duplicate samples; error bars indicate SD. **A) **Comparison of the potencies of sCD4-*35*-17b and sCD4-*40*-17b against the 91US054 pseudotype and infectious virus. In the TZM-bl system, the IC_50 _values against the pseudotype were 11 nM for both sCD4-*35*-17b and sCD4-*40*-17b; the value against the infectious virus was 22 nM for sCD4-*40*-17b. In the MAGI-CCR5 system, the IC_50 _value against the infectious virus was 30 nM. **B) **Comparison of the effects against the indicated Env pseudotypes of sCD4-*40*-17b, sCD4-*5*-17b (shorter linker) and proteins representing individual moieties (sCD4 alone, or in combination with 17b SCFv). The IC_50 _values for sCD4-*40*-17b were 12.3 nM against CAAN5342.A2, 9.8 nM against RW020, and 0.8 nM against YU2; the value for sCD4-*5*-17b against YU2 was 16.5 nM.

One major focus was to test whether lengthening the L1 linker might convey greater neutralization breadth to sCD4-17b. Fig. 2A shows results in the TZM-bl assay with pseudotype virus of the primary isolate US054 (clade B), which was insensitive to sCD4-*35*-17b in the previously reported MAGI-CCR5 assay [29]. Perhaps surprisingly, both the original sCD4-*35-17b *and the new variant sCD4-*40*-17b neutralized effectively and with equivalent potencies (IC_50 _= 11 nM for each). As a negative control, no neutralizing activity was observed against pseudotype particles bearing the envelope glycoprotein of amphotropic murine leukemia virus (data not shown).

Since the previous MAGI-CCR5 assays demonstrating sCD4-17b resistance of several HIV-1 isolates were performed with infectious virus rather than Env pseudotypes [29], we compared both particle types in the TZM-bl assay, again examining the 91US054 primary isolate. As shown in Fig. 2A, sCD4-*40*-17b neutralized infectious virus with potency (IC_50 _= 22 nM) similar to that for pseudotyped particles. Additional experiments assaying infectious virus preparations in the TZM-bl assay indicated neutralization of several other primary isolates previously found to be resistant in the MAGI-CCR5 assay (93IN905, clade C; 93TH073, clade E; 93BR029, clade F) [29], with indistinguishable potencies for sCD4-*35*-17b and sCD4-*40*-17b (data not shown). We also examined whether features of the MAGI-CCR5 assay might be responsible for the previously reported resistance of some HIV-1 primary isolates to sCD4-17b. In the present study, the highest concentration (92 nM) of affinity purified sCD4-17b protein used was ~3-fold higher than the maximum concentration (32 nM) of unpurified protein used in our earlier report. As shown in Fig. 2A, sCD4-*40*-17b effectively neutralized infectious virus of the US054 strain in the MAGI-CCR5 assay, albeit with a somewhat weaker potency (IC_50 _= 30 nM) compared to the TZM-bl assay. We conclude that the previously described insensitivity of some HIV-1 primary isolates to sCD4-17b was likely due to a combination of factors including insufficient concentrations of the inhibitor and use of unpurified protein, rather than to an insufficiently long L1 linker or to the use of infectious virus in the previous study. Additional experiments (see below) confirm the sCD4-17b sensitivity of infectious virus particles from other HIV-1 isolates.

Very different results were obtained with sCD4-*5*-17b, whose LI linker (a single G_4_S motif) is predicted to be too short to enable simultaneous binding of the sCD4 and 17b SCFv moieties to a single gp120 subunit. Fig. 2B shows that for the primary isolates CAAN (clade B) and 92RW020 (clade A), the IC_50 _values for sCD4-*40*-17b were in the range of 10 nM, but negligible inhibition occurred over the same concentration range with sCD4-*5*-17b or with sCD4 alone or in combination with equimolar concentrations of unlinked17b SCFv. When even more sensitive isolates were examined, a more subtle distinction emerged, as shown for the YU2 primary isolate (clade B). Very potent neutralization occurred with sCD4-*40*-17b (IC_50 _0.8 nM), whereas no inhibition was observed over the same concentration range with sCD4, alone or in combination with unlinked 17bSCFv; however sCD4-*5*-17b neutralized, albeit with a 20-fold weaker potency (IC_50 _16.5 nM) compared to sCD4-*40*-17b. Similar findings were observed with several other highly sensitive isolates (e.g. 93IN905, Clade C primary isolate, data not shown). The experiments presented in the following sections were performed with sCD4-*40*-17b.

### Extremely broad and potent activity of sCD4-17b against Env pseudotypes from genetically diverse primary isolates

**Figure 3:**
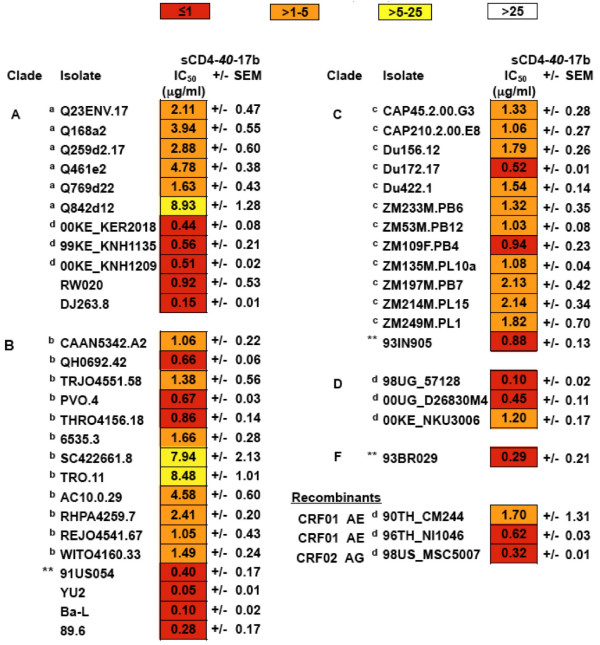
**Breadth of sCD4-*40*-17b activity against pseudotypes from genetically diverse HIV-1 primary isolates**. Nearly all entries are primary isolates for which the Env sequences were originally obtained by direct cloning from infected tissue; the exceptions are the laboratory-adapted Ba-L, and 89.6 strains. The IC_50 _values (μg/ml) represent the mean of multiple independent assays (+/- SEM) (3-5 replicate assays in most cases, 2 in a few instances). The values are color coded according to the IC_50 _ranges as indicated at the top of the figure, with red<orange<yellow<white. Note that none of the IC_50 _values are in the white range (least potent). The superscript letters indicate the references describing the individual isolates, as follows: ^a^[41], clade A; ^b^[39], clade B, tier 2 reference panel; ^c^[40], clade C reference panel; ^d ^[V. Polonis and S. Tovanabutra, personal communication], clades A, D, AE and AG. Further details on various features of these isolates can be found in these cited references. ** Indicates isolates previously found to be insensitive to sCD4-17b in the MAGI-CCR5 assay using infectious virus [29].

Genetically diverse HIV-1 isolates from different geographic regions worldwide share the requirement for both CD4 and coreceptor (CCR5 and/or CXCR4) as target cell receptors for virus entry. The 17b epitope on the bridging sheet component of the gp120 coreceptor binding site has been shown to be highly conserved on most/all HIV-1 strains examined [1,4,20,42], leading us to predict that the neutralizing activity of sCD4-17b should be extremely broad. Fig. 3 shows that this is indeed the case, based on analysis of nearly 4 dozen pseudotypes bearing Envs from each of the major genetic subtypes representing clades A, B, C, D, F, and the circulating recombinant forms CRF01_AE and CRF01AG. Based on categorization of potency previously employed by other groups assessing sensitivities of Env pseudotypes to specific MAbs in the TZM-bl assay [39-41] [also V. Polonis and S. Tovanabutra, personal communication], virtually every one of the pseudotypes examined was neutralized by sCD4-*40*-17b; in fact nearly all IC_50 _values were in the ≤ 1 μg/ml or >1-5 μg/ml categories, with only 3 in the >5-25 μg/ml category (actually <10 μg/ml). Of particular note, each of the newly tested isolates previously described as sCD4-17b-insensitive in the MAGI-CCR5 assay [29] (91US054, 93IN905, and 93BR029) was extremely sensitive (IC_50 _≤ 1 μg/ml).

**Figure 4:**
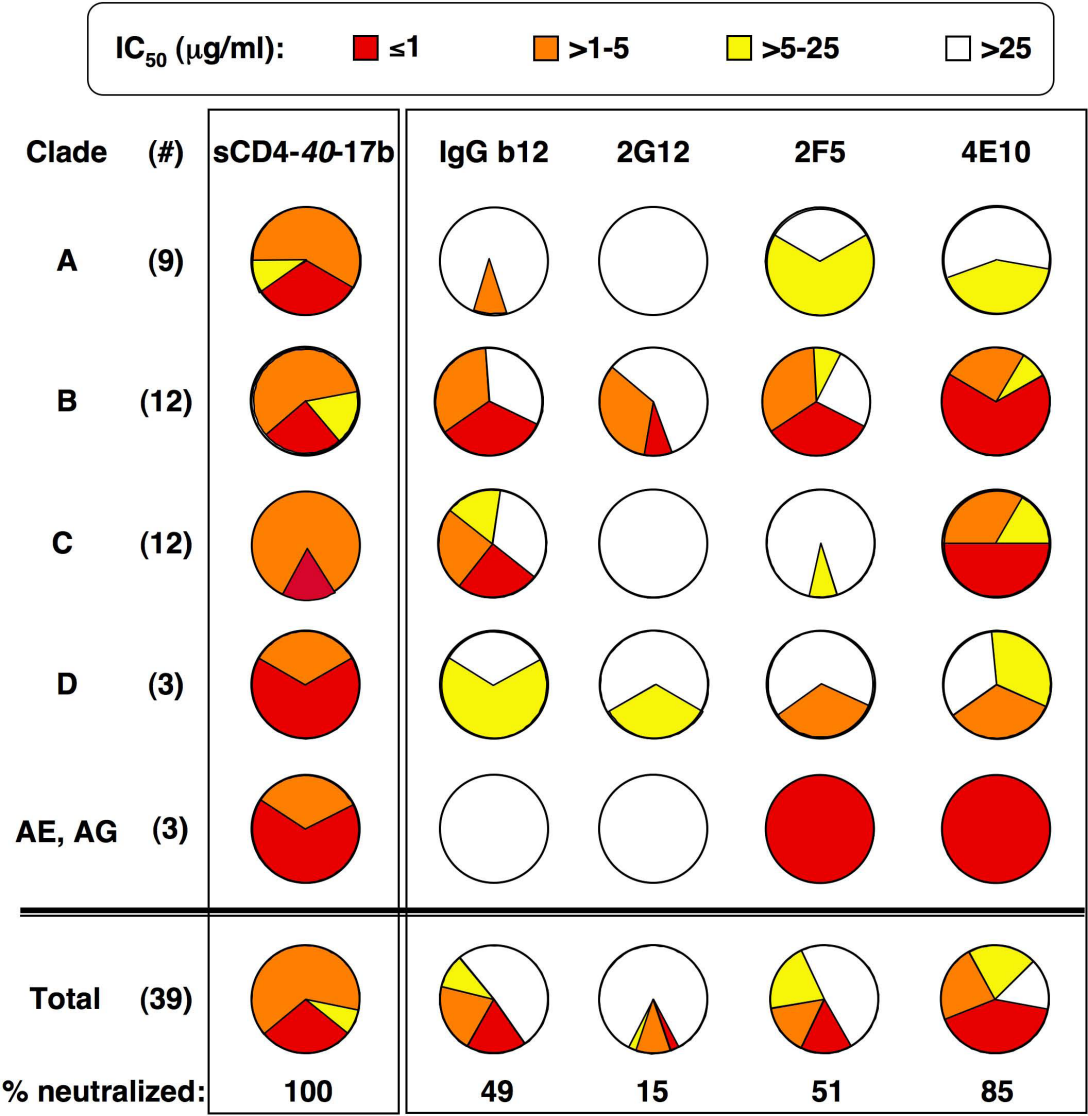
**Comparison of neutralization breadth of sCD4-*40*-17b with broadly neutralizing monoclonal antibodies**. Each pie graph shows the fraction of isolates within each IC_50 _range for each agent tested against isolates from the indicated clades. The color-coding for IC_50 _ranges (indicated at the top of the Figure) is the same as in Figure 3, with red<orange<yellow<white. Note that whereas for sCD4-*40*-17b none of the clades had isolates with IC_50 _values in the white range (least potent), each of the MAbs had some or all isolates with IC_50 _values in the white range for at least some clades; thus when the total isolates where evaluated for "% neutralized", the value was 100 for sCD4-*40*-17b but considerably less for the MAbs. The pie graphs are based on the indicated number of isolates (#) in each clade, which are the ones designated in Figure 3 with superscript letters. The sCD4-*40*-17b results are based on the data in Fig. 3; the MAb results are based on findings by others as follows: clade A, 6 isolates [41], 3 isolates [V. Polonis and S. Tovanabutra, personal communication]; clade B, all 12 isolates (reference panel clade B) [39]; clade C, all 12 isolates (reference panel clade C) [40]; all 3 clade D isolates [V. Polonis and S. Tovanabutra, personal communication]; all 3 clade AE and AG isolates [V. Polonis and S. Tovanabutra, personal communication].

This breadth is particularly impressive when compared with sensitivities to the well-characterized broadly neutralizing MAbs IgG b12, 2G12, 2F5 and 4E10. Fig. 4 shows such comparisons for the subset of pseudotypes from Fig. 3 that have been analyzed in detail by others for sensitivity to these MAbs; these include 9 from clade A [41] [also V. Polonis and S. Tovanabutra, personal communication], 12 each from clade B [39]) and clade C [40] (both sets selected as reference panels for vaccine evaluation), 3 from clade D and 3 from recombinant clades CRF01_AE and CRF01AG (V. Polonis and S. Tovanabutra, personal communication). The pie graphs in Fig. 4 highlight the significantly greater breadth across all clades of sCD4-*40*-17b compared to the activities of each of these broadly neutralizing MAbs, all of which were ineffective against multiple isolates from several clades (based on data from others, and confirmed by us in a limited number of cases, data not shown).

### Neutralization of HIV-1 by sCD4-17b: independence from cellular source of virion particles

**Figure 5:**
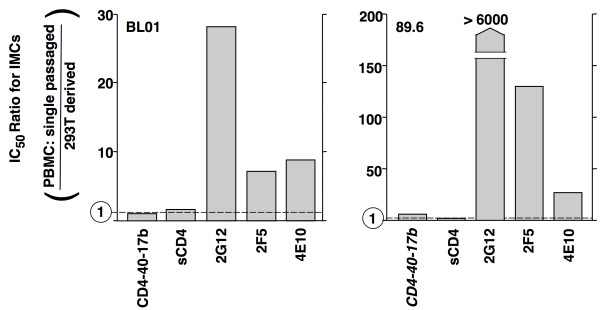
**HIV-1 sensitivity to sCD4-*40*-17b: independence from cellular source of virions**. IMCs from strains BL01 and 89.6 were used to generate virus particles directly from transfected 293T cells; a second set of viruses was generated by single passage of the 293T-derived particles in PBMCs [30]. Both sets of particles (directly provided to us by M. Louder and J. Mascola) were analyzed for sensitivity to the agents shown. The results are plotted as the IC_50 _ratios of the PBMC-passaged to the 293T-derived virus particles. In each graph, the dotted line indicates a ratio of 1 (i.e., equal potency against each type of virus particle).

The neutralization sensitivity of HIV-1 can depend strongly on the cell type from which the virus particles are produced. A particularly clear demonstration of this involved analyses of IMCs from which virions were generated directly from plasmid-transfected 293T cells versus after a single passage of the 293T-derived virions through mitogen-stimulated PBMCs. Despite verification of complete Env sequence identity between the matched pairs of virions, the broadly neutralizing MAbs were found to be significantly less potent against the PBMC-derived virions compared to their cell line-derived counterparts [30]. We assessed the activity of sCD4-*40*-17b against matched pairs of 293-derived vs. PBMC-single passaged virions; the activities of four broadly neutralizing MAbs were tested for comparison. Fig. 5 shows results with IMCs from two distinct clade B isolates, BL01 and 89.6; the results are expressed as the IC_50 _ratios of PBMC-derived versus 293 cell-derived virions. While our results verified the previous report [30] that PBMC-passage virions were considerably less sensitive than their 293-derived counterparts to neutralization by the MAbs, we found that they were comparably sensitive to sCD4-*40*-17b. Sensitivity to sCD4 was also independent of the cellular source of the virions, consistent with previously reported findings [30].

## Discussion

The data presented herein confirm and extend our previous conclusion [29] that the neutralizing potency of sCD4-17b derives from the ability of both moieties on a single bifunctional molecule to associate simultaneously with their corresponding binding sites on a single gp120 subunit. Variants of the protein with sufficiently long L1 linkers, i.e. the original sCD4-*35*-17b and the newly described sCD4-*40*-17b displayed potency much greater than either sCD4 alone, or in equimolar amounts with unlinked 17b SCFV. Preliminary results indicated that a construct containing an L1 linker of 55 amino acids (11 G_4_S repeats) was comparably effective (data not shown). By contrast, sCD4-*5*-17b, which contains an L1 linker too short to allow simultaneous binding of the sCD4 and 17b moieties, had a much weaker potency. However when tested against strains that were highly sensitive to sCD4-17b, sCD4-*5*-17b proved significantly more effective than the mixture of unlinked sCD4 plus 17b SCFv. A plausible explanation is that while sCD4-*5*-17b was incapable of mediating simultaneous binding of both components to the same gp120 subunit, reversible binding of molecules via the sCD4 portion effectively increased the local concentration of 17b SCFv in the vicinity of Env, where it could bind to gp120 subunits that had been induced by sCD4 moieties on separate molecules of the chimeric protein. Thus bifunctional binding molecules can potentially display enhanced activities even when the two binding moieties are incapable of interacting simultaneously with a single target molecule.

The exceptional breadth displayed by sCD4-17b (neutralization of 100% of isolates tested from genetically diverse HIV-1 subtypes) confirms our original expectation, based on the requirement for CD4 binding amongst all natural HIV variants coupled with the high conservation of the bridging sheet due to its critical role in coreceptor binding of HIV-1 (and HIV-2 as well [16]). The breadth exceeded by a considerable margin those of the well-characterized broadly neutralizing MAbs IgG b12, 2G12, 2F5 and 4E10 tested against the same HIV-1 isolates [39,40,43] (also V. Polonis and S. Tovanabutra, personal communication). Of interest in this regard is the recent report of 2 new human gp120-targeted MAbs, PG9 and PG16, that neutralize a large fraction (79% and 73%, respectively) of Env pseudotypes from a genetically diverse panel of primary HIV-1 isolates (IC_50 _values ranging from 0.001 to 50 μg/ml); of the 162 pseudotypes examined, only 32 were resistant to both MAbs (IC_50 _>50 μg/ml) [44]. In the present study, 1 of these PG9/PG16-resistant isolates was tested against sCD4-17b and was found to be highly sensitive (QH0692.42, Clade B, IC_50 _= 0.66 μg/ml, Fig. 3). It will be most interesting to test the sCD4-17b sensitivities of other strains resistant to these newly described MAbs.

Our previous observation of limited breadth in the MAGI-CCR5 system was not due to the requirement of a longer L1 linker for some strains, as we previously speculated. Nor was it associated with the use of infectious virus in that system, since the TZM-bl assay demonstrated sCD4-17b sensitivity for infectious virus from several isolates (Fig. 2A, Fig. 5); where tested, the potency was comparable to that observed with the corresponding Env pseudotyped particles (Fig. 2A). Retesting one of the previously described resistant isolates, namely 91US054, in the MAGI-CCR5 assay suggested that the limitation of neutralization breadth in our earlier study might have resulted from insufficient concentration of sCD4-17b (unpurified or partially purified), coupled with the higher virus input volume required in the MAGI-CCR5 assay compared to the TZM-bl assay (20 μl versus 5 μl, respectively). We believe the present findings of extremely broad neutralization activity of sCD4-17b in the commonly used TZM-bl assay override the limitations noted in our previous report, which were most likely due to the specific conditions associated with those experiments rather than to inherent properties of the sCD4-17b protein or the Env glycoproteins that it targets.

The HIV-1 IMC experiments demonstrated a marked contrast between sCD4-17b and neutralizing antibodies with respect to the influence of the cellular source from which the virus particles were derived. The potency of sCD4-*40*-17b was equivalent against virions isolated directly from the transfected producer cell line and progeny virions obtained after single passage through PBMCs (Fig. 5). By contrast, the PBMC-passaged virions were significantly less sensitive to the broadly neutralizing MAbs, as previously shown by the group that provided the IMCs for our experiments; importantly, they also demonstrated the absence of any sequence change in Env after PBMC passage [30]. In that earlier report, several possible explanations were offered for the reduced antibody susceptibility of PBMC-passaged viruses compared to their cell line-derived counterparts, including higher levels of Env on virions from PBMCs, differential incorporation of host cell factors such as adhesion proteins during virus assembly in PBMCs, and producer cell-dependent biochemical differences in the gp160, such as differential glycosylation. In our opinion, the first two mechanisms might be expected to result in neutralization differences that are similar for various classes of Env-blocking agents, and thus would not readily explain the lack of effect of sensitivity to sCD4-17b and sCD4 despite the strong effects on neutralizing antibody sensitivity. Differences in glycosylation seem an appealing possibility for several reasons. First, the decreased antibody sensitivity upon PBMC passage was most dramatic for the 2G12 MAb (Fig. 5, also [30]), whose epitope on gp120 consists of large mannose-rich carbohydrate clusters that can be recognized by an unusual domain-swapped structure of the antibody [45]. Second, gp120 is noted for its extensive "glycan shield' that continually evolves in the infected host to sterically block neutralizing antibodies directed against non-carbohydrate epitopes without affecting receptor binding [46], since the CD4 binding site and the bridging sheet are devoid of carbohydrate [10]. Indeed there is precedence for variations in glycosylation patterns of isogenic HIV-1 Envs dependent on the producer cell type [47]. The potent activity of sCD4-17b against PBMC-produced virions is critical, since these presumably reflect the properties of *in vivo *particles more closely than do the cell line-derived virions.

The broad, potent antiviral activity of sCD4-17b suggests several possible applications. Passive immunotherapy with MAbs has been examined both in nonhuman primate models and human clinical trials [48], and sCD4-17b could be considered as an additional component to a mixture of broadly neutralizing MAbs; however the practical limitations of passive immunotherapy for treating chronic HIV infection greatly reduces enthusiasm for this mode of use. A related alternative would involve gene therapy strategies using either viral vectors [49] or engineered hematopoietic stem cells [50] to continually produce sCD4-17b in the body, for treatment or protection against HIV infection. For such applications, it is likely that the molecule would need to be modified for enhanced plasma half-life by linking it to immunoglobulin constant regions, as has been done for sCD4 [51]; indeed such a modification might provide the additional advantage of increased potency due to multivalent binding. Perhaps a more likely antiviral application of sCD4-17b would be as a topical microbicide to prevent sexual transmission. Several classes of proteins and peptides targeting either the virus or receptors on the host cell are being actively studied for this purpose [52,53], encouraged by technologies to manufacture candidate proteins on an economically viable scale [54]. A particularly intriguing approach involves genetic modification of commensal bacteria native to the healthy vaginal or rectal mucosa to produce the anti-HIV proteins *in situ *[55]. For example, vaginal strains of *Lactobacillus *producing CD4 in either secreted [56] or surface-bound [57] forms have been described; this "live microbicide" concept is being investigated with various potent anti-HIV proteins and peptides [58-60], and preliminary efforts have been undertaken for sCD4-17b (L. Lagenaur and E. Berger, unpublished). For any of the applications suggested above, sCD4-17b has significant advantages compared to some other candidate proteins in that it is highly specific for HIV, and is composed of entirely human-derived sequences (except for the linkers). Thus problems associated with immunogenicity and induction of inflammatory responses are predicted to be relatively minor. We propose that sCD4-17b warrants continued investigation in the ongoing efforts to develop new antiviral strategies to combat the HIV/AIDS pandemic.

## Conclusions

sCD4-17b neutralizes HIV-1 with high potency and great breadth against genetically diverse primary isolates. It is equivalently active against virus particles generated from different producer cell types (cell line versus PBMC). These results support the continued investigation of various modalities by which sCD4-17b can be employed against HIV infection.

## Competing interests

EB is co-inventor on an NIH-owned patent for sCD4-17b.

## Authors' contributions

LL, VV, BD, and EB contributed to the conception and design of studies. LL, VV, BD, and VB contributed to the conduct experiments and analysis of data. EB contributed to the initial drafting and writing of the manuscript.
